# The most used medicinal plants by communities in Mahaboboka, Amboronabo, Mikoboka, Southwestern Madagascar

**DOI:** 10.1186/s13002-017-0147-x

**Published:** 2017-03-09

**Authors:** Tabita N. Randrianarivony, Aro Vonjy Ramarosandratana, Tefy H. Andriamihajarivo, Fortunat Rakotoarivony, Vololoniaina H. Jeannoda, Armand Randrianasolo, Rainer W. Bussmann

**Affiliations:** 1 Missouri Botanical Garden, Madagascar Research and Conservation Program, BP 3391, Antananarivo, 101 Madagascar; 2 Plant Biology and Ecology, University of Antananarivo, BP 906, Antananarivo, 101 Madagascar; 3 William L. Brown Center, Missouri Botanical Garden, P.O. Box 299, St. Louis, MO 63166-0299 USA

**Keywords:** Medicinal plants, Traditional knowledge, Arid areas, Healthcare

## Abstract

**Background:**

This paper reports a study undertaken in three remote communities (Mahaboboka, Amboronabo, Mikoboka), located in Sakaraha, Southwestern Madagascar. Not only villages are far away from sanitary infrastructures and doctors but drugs and consulting fees are unaffordable to villagers. They rely essentially on natural resources for health care as for most of rural areas in Madagascar. This paper aims to document medicinal plants used by communities in Sakaraha and to present the most important plant species used in traditional medicine.

**Methods:**

Semi – structured interview was conducted within 214 informants in 34 villages of the study area. Different ailments encountered in the site study were classified in various categories. For data analysis, frequency of citation (Fq), Informant Consensus Factor (Fic), Fidelity Level (FL) and Use Value (UV) were assessed to find agreement among informants about the use of plants as remedies. Mann-Whitney, Kruskall-Wallis and Spearman correlation tests were performed to determine use of medicinal plants following social status of informants.

**Results:**

A total of 235 medicinal plant species belonging to 198 genera and 75 families were inventoried. The richest families in species used for medicinal purposes were: Fabaceae, Apocynaceae, Rubiaceae, Euphorbiaceae, Asteraceae, and Poaceae. Plant species cited by informants were used to treat 76 various ailments classified in 13 categories. Leaves and leafy twigs were the most used plant parts and decoction was the mostly cited way of preparation of these medicinal plants species. In average, local people cited 6.7 ± 6.03 medicinal taxa among them, *Cedrelopsis grevei* is the most cited medicinal plants (Fq. 0.28). With *Cedrelopsis grevei* (UV = 0.48), *Henonia scoparia* (UV = 0.43) are mostly used species. *Leonotis nepetifolia* (FL = 96%) and *Strychnos henningsii* (FL = 92%) are plant species claimed by high percentage of informants to treat the Digestive System Disorder.

**Conclusions:**

This study highlighted that medicinal plants used by people from three communities in the Southwestern Madagascar are diverse. These plants species ensure care to all family members including babies, children, mothers and adult people. Through this study, newly reported medicinal plants were identified for further work.

**Electronic supplementary material:**

The online version of this article (doi:10.1186/s13002-017-0147-x) contains supplementary material, which is available to authorized users.

## Background

Madagascar hosts one of the richest natural heritage in the world but is classified among the least developed countries with low Gross Domestic Product (GDP) per capita estimated at 409$ in 2015 (http://www.tradingeconomics.com/madagascar/gdp-per-capita-ppp). This poverty contributes to a rapid loss of biodiversity in a country, where exploiting natural resources are the unique available sources of incomes for most of people living in rural areas. Due to health facilities that do not meet standards, together with poor sanitary infrastructures and unmotivated medical staff [1], unaffordable drug costs and high consulting fees, use of medicinal plants is now often part of the first resort delivered and the only accessible therapy to people from several localities in Madagascar [2–4] including communities from remote areas like Mahaboboka, Mikoboka and Amboronabo.

In many Malagasy societies, apart from simple diseases like fever, cold, injury and burn, most are believed to come from unnatural sources, superstition and religious conviction. Simple diseases are treated by elders and stay at the family level, the unnatural ones needed helps of spirit healers. Traditional healers are called in different ways according to their ethnic group, their region or their ways of healing for example, “*Mpanazary*” (sorcerer in Betsimisaraka ethnic group), “*Mpitana*” (guardian of talismans for Merina ethnic group), “*Ombiasy*” (Spirit healers in the southern Madagascar) [5]. They proceed differently. Some ask guidance of spirits to reveal causes of diseases, “*Tromba*” [6], others deliver the sick people from spirit that obsesses them, “*Bilo*” [7] and some use divinations, they are called spirit healers. Other traditional healers use only plants and called herbalists. Some use massage, and saliva to cure diseases. In general plants accompany those different process of traditional healing.

During the Malagasy monarchy, the use of plants was very common, but it has been banned and progressively replaced by modern medicine during the colonial era [8, 9]. Later on, Professor Ratsimamanga, founder of the Malagasy Institute of Applied Research (IMRA) in 1958, brought back the important value of plants in healing, and studied the chemical compounds of some of the plants used in traditional medicine [10]. Since then, ethnobotany of medicinal plants were rediscovered, studied, published by scholars, and improved remedies developed from traditional medicine were successfully marketed. The Malagasy Health Ministry officially recognized the traditional medicine and they integrated the Traditional Health Practitioner Association, created in 2002 [11], in the conventional national health system. Nowadays people from rural and some from urban areas consider one more time the use of plants. However, even if monographs of medicinal plants of some areas such as Alaotra-Mangoro, Ambongo-Boina, Antakarana, Toliara II, have been published [12–15], medicinal uses of plants from other regions are still not systematically investigated. Moreover, most of medicinal plants studies in Madagascar were focalized on one plant species or one illness. And when ethnobotanical studies touched an entire area, it did not consider forest conservation and social aspects like we did within this work.

Because of the rapid destruction of plant species habitat, there is a race against time to integrate on traditional knowledge around the world and mostly in developing country such as Madagascar where man pressure is very high. Inventory of indigenous knowledge on plant uses has been found to be important for species management [16, 17]. This study is part of the conservation plan of Analavelona sacred forest located in South-western Madagascar, to list plant uses for a better management of forestry resources. This paper aims to document medicinal plants used by communities in Sakaraha and to present the most important plant species used in traditional medicine in these communities. For this study, we hypothesized that (1) people have greater agreement to the category of illnesses related to malaria, wounds, diarrhea and dentistry diseases, which are prioritized by the World Health Organization (WHO) in Madagascar, (2) women cited more medicinal plant for diseases related to children.

## Methods

### Study area and demography

**Fig. 1:**
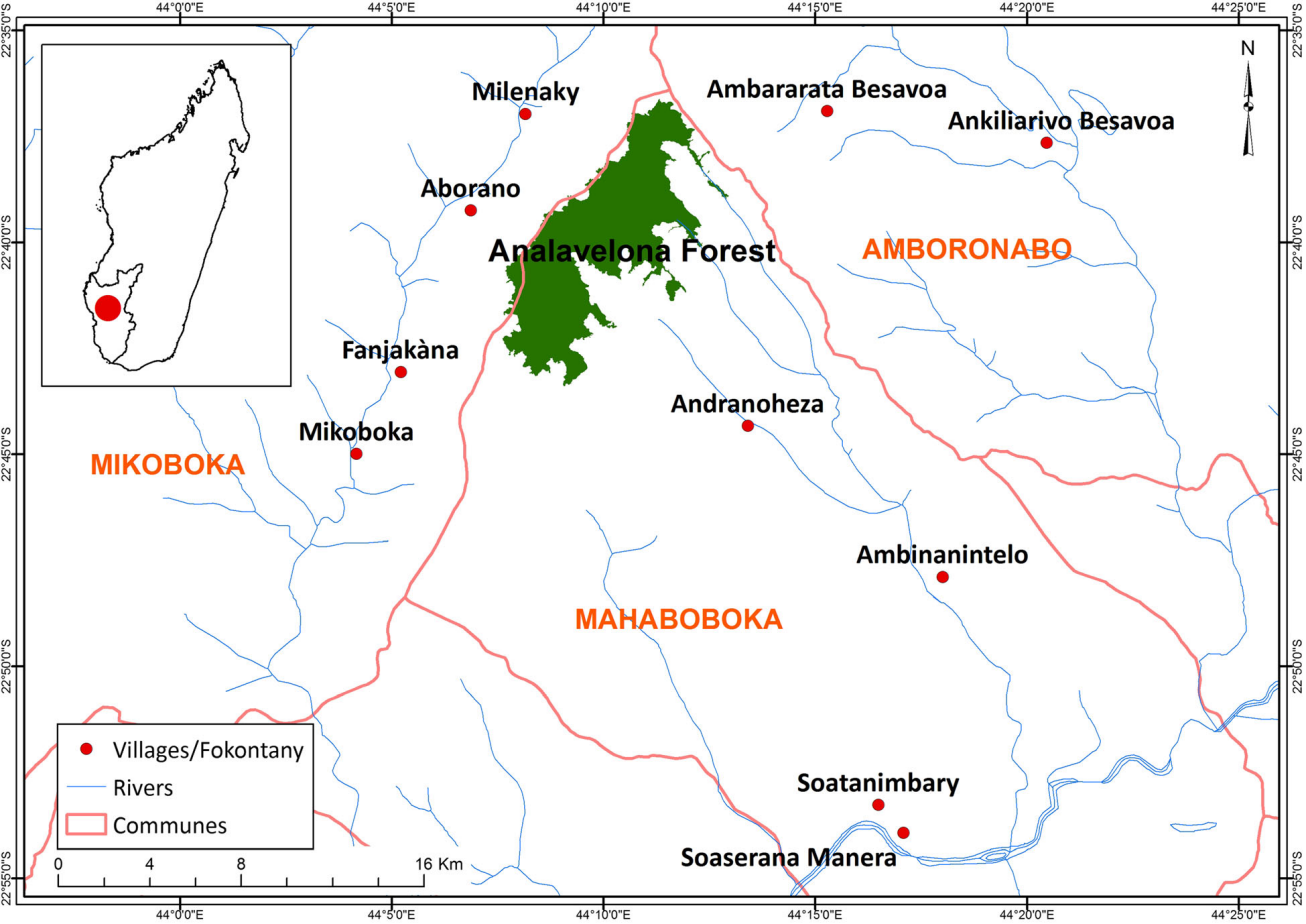
Localization of the study area

The study area covered 34 villages belonging to the three communities (a territorial administrative entity lead by mayor, below district entity) of Mahaboboka, Amboronabo and Mikoboka, 30 km in the South-west of Sakaraha district. The study site is located in arid area in the Southwestern Madagascar and situated between 22°36′ and 22°54′ south latitude and between 44°02′ and 44°24′ east longitude with an altitude ranging from 400 to 1350 m (Fig. 1). Site topography is heterogeneous and characterized by a vast plain that extends from Andranoheza valley to the Fiherenana river, mountains including Analavelona massif and the “*Arorà*” lake in Mitsinjorano. The soil is typically ferruginous but Analavelona forest sits on volcanic soil corresponding to basalt [18]. The annual average of temperature is 20°C and annual rainfall is between 750 and 1000 mm [19]. The vegetation is typically of dry areas of Southwestern Madagascar with savannah and dry forests. In this area, the sub-humid forest of Analavelona is an exception where 403 plant species grouped in 100 families were inventoried and 73% of them are endemic to Madagascar [20]. Villagers surrounding Analavelona forest and even spirit healer from far away have traditional rights to access to medicinal and magical plants species from the sacred forest following local rules [21].

Population living around Analavelona forest is estimated at 29,833 with a density of 12 people per km^−2^ [20]. In 2014, only three doctors, two midwives and nine sanitary agents were in charge of all communities at four health centers. Villagers are composed of nine ethnic groups; Bara are the most abundant and dominant followed by Antanosy, Antandroy, Masikoro, Vezo, Sakalava, Mahafaly, Bestileo and Antaisaka [22]. The chief of village locally known as “*Lonaky*” is an authority person to the communities, they are the keeper of plant knowledge that they inherit from their ancestors and spirit healers (“*Ombiasy*”). Cultivation of rice, sweet potatoes, cassava, and corn as main crops and cattle rising are the main activities of villagers. About their education index, level of literacy and numeracy is very low, only 20% of villagers are literate [22] and most of them stopped at primary school.

### Administrative authorization

Prior to any field works, details of the project including responsible of data and plants collection were submitted to the Malagasy Ministry of Environment, Ecology, and Forest (MEEF) for evaluation. Thereafter, MEEF granted a written permission to collect herbarium specimens which was stamped by the regional office of the forest department in Sakaraha. Authorization of mayors of each community were obtained after a personal visit and presentation of objectives and outcomes of the project. This was followed by signature of an authorization of conducting a field study by the president of Fokontany (a lowest administrative subdivision in Madagascar).

### Traditional and informants consent

The village chief known as “*Lonaky*” was approached and made aware of the investigation process. Schedule and timing of field work, team members including local guide and researcher were presented and introduced to the “*Lonaky*” to get his consent. Interviews with villagers were only performed once a verbal permit was obtained from the “*Lonaky*”. Traditional healers were also contacted in advance to get their verbal consent. Verbal consent of informants was requested prior to interviews, fulfilling the requirements of the seventh article of the Nagoya protocol [23].

### Sampling method

All expert informants found in visited villages, including traditional healers, village chief and elders, who were people specialist on plant uses and healing, were approached first for the survey. In total, 8 spirit healers, 4 traditional midwives, 4 president of Fokontany and 8 village chief were interviewed. These expert informants were mostly men (21 men). About laypeople, they are from different ethnic groups, gender, occupation and age and have accepted to be interviewed. The same questionnaire were used with experts and laypeople but we insisted on the way of preparation and administration, abundance of plants and their localization in the fields with expert informants.

### Ethnobotanical survey

Contacts with “*Lonaky*” and administrative authorities were established in December 2010, and surveys were conducted during 15 weeks between January 2011 and March 2012. Semi – structured interviews [24] were conducted with informants using the local dialect. A local researcher assistant was hired to facilitate the conversation to avoid any misunderstanding during interview. Informants were spirit healers (“*Ombiasy*”), village chief (“*Lonaky*”), traditional midwives, men and women aged from 14 to 93 years, living in the three communities. Field and plant interviews were performed with key informants in order to know more about medicinal plants used by local people [24]. Plant local names, medicinal uses, used plant parts, mode of preparation, mode of administration and their availability in nature were noted during interviews. Demographic data on the informants such as gender, age, ethnic group, occupation and education level were documented.

### Plant collection

As plant local names were given by informants, collection of voucher specimens related to the name given by each informant was done following plant standard collection by Dold et al. [25]. At least, three herbarium specimens were collected and vouchers were deposited at both national (Parc Botanique et Zoologique de Tsimbazaza, Antananarivo, Madagascar) and international (Missouri Botanical Garden, Saint Louis, USA; Museum National d'Histoire Naturelle, Paris, France) herbaria for plant species identification. Accepted names from the catalogue of vascular plants of Madagascar website (http://www.tropicos.org/project/mada) were used.

### Data processing

A list of medicinal plant species cited by each informant was established in Excel® sheet table. Along the list of plant taxa, the table also contains the plant local and scientific names, family names, ailments, plant part used, applications, preparation and administration mode, and type of vegetation where the plants are growing. Cited ailments were classified in different categories according to Cámara-Leret et al. [26].

#### Quantitative data processing

Calculation of frequency of citation (Fq) is a way to determine the most useful plants. Frequency of citation index can be calculated by the ratio between the number of informants who mentioned a given species and the total number of informants.

Then, agreement among informants about a particular plants for a particular remedy was determined by the Informant Consensus Factor (Fic) and Fidelity Level (FL). The most important medicinal uses of plants were assessed by the Use Value (UV) index.

The Informant Consensus Factor (Fic) is used to determine the importance of each medicinal use category depending on the homogeneity of informant’s answer [27]. It was calculated according to the formula of Heinrich et al. [28] as followed:

$$ \mathrm{I}\mathrm{F}\mathrm{C}=\frac{\left(\mathrm{Nur}\ \hbox{-}\ \mathrm{Nt}\right)\ }{\left(\mathrm{Nur}\ \hbox{-}\ 1\right)} $$

Where, Nur = number of use reports from informants for a particular plant-use category; Nt = number of taxa or species that are used for that plant use category for all informants.

The product of this factor range from 0 and 1, a highest value of Fic (close to 1) indicates a greater consensus on the use of a given plants to treat a particular ailment category. A low value of Fic (close to 0) indicates that the informants disagree with the category of use of a plant [27, 29].

The fidelity level (FL) was also calculated as a tool to get the percentage of informants claiming the use of a certain plant for the same major purpose. It is defined as the ratio between the number of informants who independently claimed a use of a plant species to treat a particular disease (Np) and the total number of informants who mentioned the plants as a medicine to treat any given disease (N) [30]:

$$ F L=\frac{Np}{\mathrm{Nx}100} $$

Plant species with high fidelity level is important to local people to treat ailments. It is noted that the number of mentions for a given plant by all of the informants for a specific disease was considered for this factor.

The use-value (UV) index was used to calculate the citation of plants during interviews [31, 32].

$$ U V=\frac{{\displaystyle \sum}\mathrm{Uis}}{\mathrm{ns}} $$

Where **∑Uis** is the sum of the total number of use citations by all informants for a given species, and **ns** is the total number of informants.

In statistics, the non-parametric tests of Mann-Whitney [33] and Kruskal-Wallis [34] were performed to assess whether or not significance difference exist in medicinal plants cited between the two genders, the two function of informants and different ethnic groups. The correlation coefficient of Spearman was calculated to elucidate if there is a correlation between informants’ age and education level and their knowledge on medicinal plants.

In the discussion part, the Jaccard similarity index (JI) was calculated to compare the results of this study with those presented with studies carried out in the southern part of Madagascar [15, 35]. We used the following formula:

$$ J I = \frac{Nc}{Na+\mathrm{N} b-\mathrm{N} c} $$

With Na : Number of medicinal plant taxa listed in this study ; Nb : Number of medicinal plant taxa listed in other study in southern Madagascar [15, 35] ; Nc : Number of medicinal plants taxa intersecting with both studies.

## Results

### Social status of informants

Male and female participants were equally distributed among the 214 informants of the study. Informants were aged from 14 to 93 years; 13.5% (*n* = 28) were above 60-year-old and 2% (*n* = 3) were under 15-year-old. Simple informants or laypeople including farmers, private and public workers, were 89% of respondents whereas 11% of them were expert informant. Regarding the ethnic group, 73.5% of informants were Bara, followed by Antanosy (17%). The majority of informants (92%) were illiterate and only few of them reached the high school diploma level (Table 1).

**Table 1:** Demographic profiles of informants in the Sakaraha district

	Description	Frequency
Gender	Male	107 (50%)
	Female	107 (50%)
Age	<15	5 (2%)
	[15–30[	63 (31%)
	[30–45[	61 (29.5%)
	[45–60[	49 (24%)
	[60–75[	22 (11%)
	≥75	6 (2.5%)
Ethnic group	Antandroy	6 (2.5%)
	Antanosy	35 (17%)
	Bara	155 (73.5%)
	Mahafaly	2 (1%)
	Masikoro	6 (2.5%)
	Mixed	6 (2.5%)
	Vezo	1 (0.5%)
Occupation	Informant expert	24 (11%)
	Simple informant	190 (88%)
Educational level	Literate	17 (9%)
	Illiterate	195 (91%)

### Ailments treated with plants and informants consent

All diseases, described in 76 indications including livestock’s diseases, were treated with plants. These indications embraced both men and women, little babies to elders. They were sorted in 13 categories such as: Blood and Cardio-Vascular Problem (BCVP), Cranial System (CS), Dental Health (DH), Digestive System Disorder (DSD), General Ailments (GA), Infectious Diseases (ID), Muscular Skeletal System (MSS), Nervous System (NS), Pregnancy, Birth and Puerperium (PBP), Reproductive System (ReprS), Respiratory System (RespS), Sensory System (SS) and Veterinary (Vet). Some of diseases like dizziness during pregnancy, or undefined pain in stomach are considered as unnatural ones so use of divination was added with plants.

The group of Digestive System Disorder was the most cited category (67%) followed by Pregnancy, Birth and Puerperium (53%). Categories of Cardio-Vascular Problem, Dental Health, Sensory System and Veterinary were the least mentioned by informant, with less than 5% of citation (Table 2).

**Table 2:** Frequency and Informant Consensus Factor of each category of illness

Category of illnesses (list of diseases)	Number of plant cited	Number of informants citing the category	Frequency of citation (%)	Informant Consensus Factor (IFC)
Blood and Cardio-Vascular System (BCVS): Cardiac problems in children, low blood pressure	3	2	1	0
Cranial System (CS): Early and late closing of baby’s fontanel	54	68	32	*0.79*
Dental Health (DH): Caries, causes teeth nerves insensitivity, dental abscess syndesmotome	14	10	5	0.07
Digestive System Disorder (DSD): Carminative, colic, diarrhea, constipation, anti-emetic, indigestion, liver disorders, intoxication from meat eating, laryngitis, gastric ulcer, intestinal ulcer, orexigenic after diarrhea, intestinal pain, dysentery	93	143	67	0.74
General ailments (GA): Weakness, headache, fever, side stitch, Yellow fever	81	93	43	0.59
Infectious Diseases (ID): Malaria, measles, scabies, tetanus, infected and syphilitic wounds, bilharzia	66	45	21	0.71
Muscular-Skeletal System (MSS): twists, fractures, low back pain, muscle aches, sprains, broken member	23	37	17	0.68
Nervous System (NS): Calming nerves, epilepsy, nerves swelling	69	41	19	0.71
Pregnancy, Birth and Puerperium (PBP): Menstrual pain, contraception, infertility treatment, pain and dizziness during pregnancy, prenatal care, induce labor, post partum recovery, healing wound after delivery, post partum hemorrhage, remove rest of placenta in uterus, promote lactation	100	114	53	*0.86*
Reproductive System (ReprS): Painful menstruation, sexually transmitted diseases (syphilis and gonorrhea), aphrodisiacs,contraceptive	11	27	13	0.57
Respiratory System (RespS): Flu, cold, bronchitis, asthma, pulmonary infection, bronchitis, cough	42	84	39	*0.77*
Sensory System (SS): Eye infections, conjunctivitis, mouth infection, boils, eye stye	6	10	5	0.55
Veterinary (Vet): Treatment of cattle’s diseases	5	6	3	0.20

In Italic are the top 3 categories of illness with high Fic value

The Fic value ranged from 0 to 0.86. Consensus of informants was low (Fic < 0.25) for plants used as a remedies for dental health, low blood pressure problem, infant heart disease and livestock’s diseases. However, high consensus were obtained in medicinal plants used for Pregnancy, Birth and Puerperium, Cranial System and Respiratory System (Table 2).

### Diversity of medicinal plants, life form and habitat

**Fig. 2:**
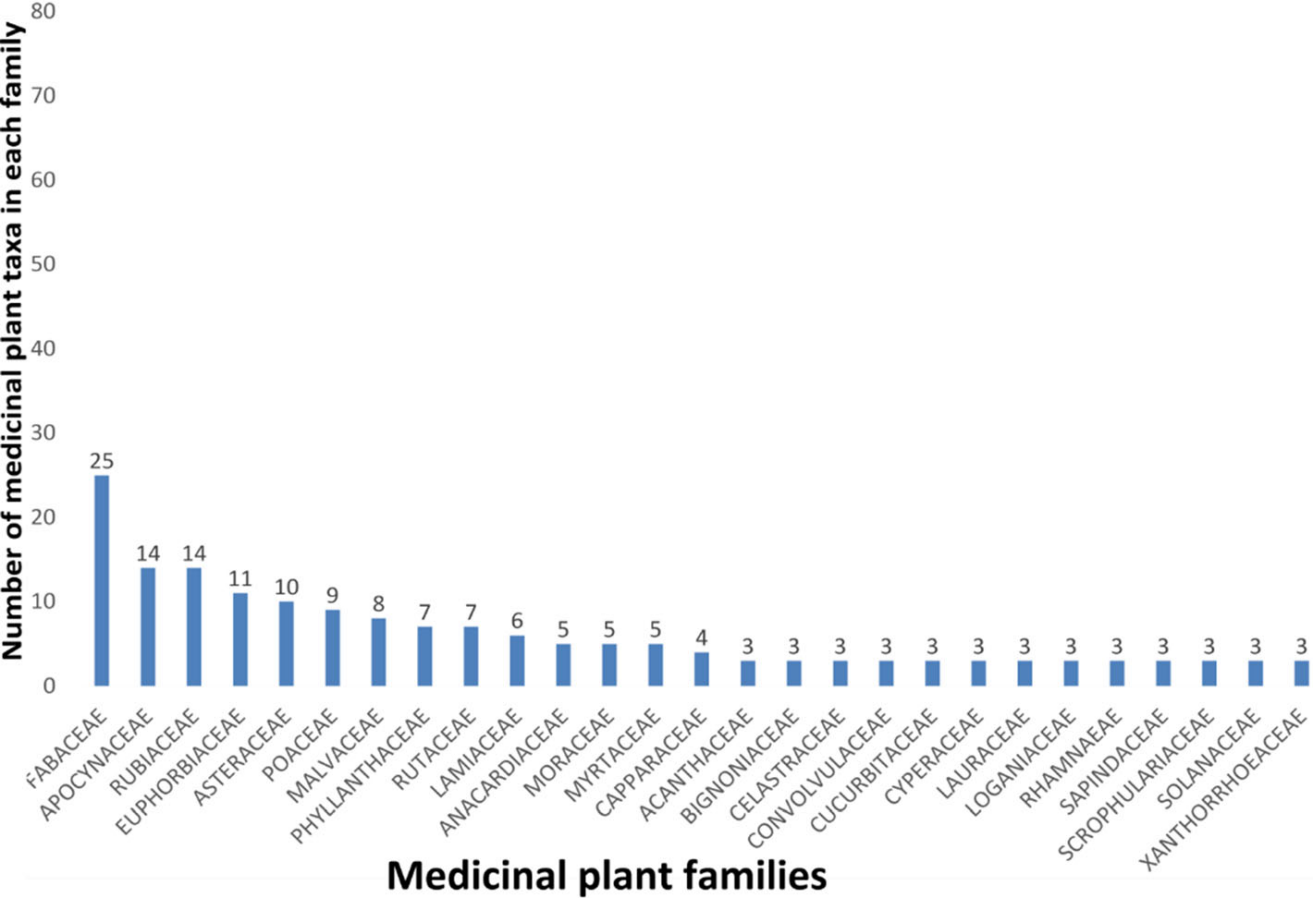
Number of useful medicinal plant species per family from the three communities in Sakaraha, South-western Madagascar

**Fig. 3:**
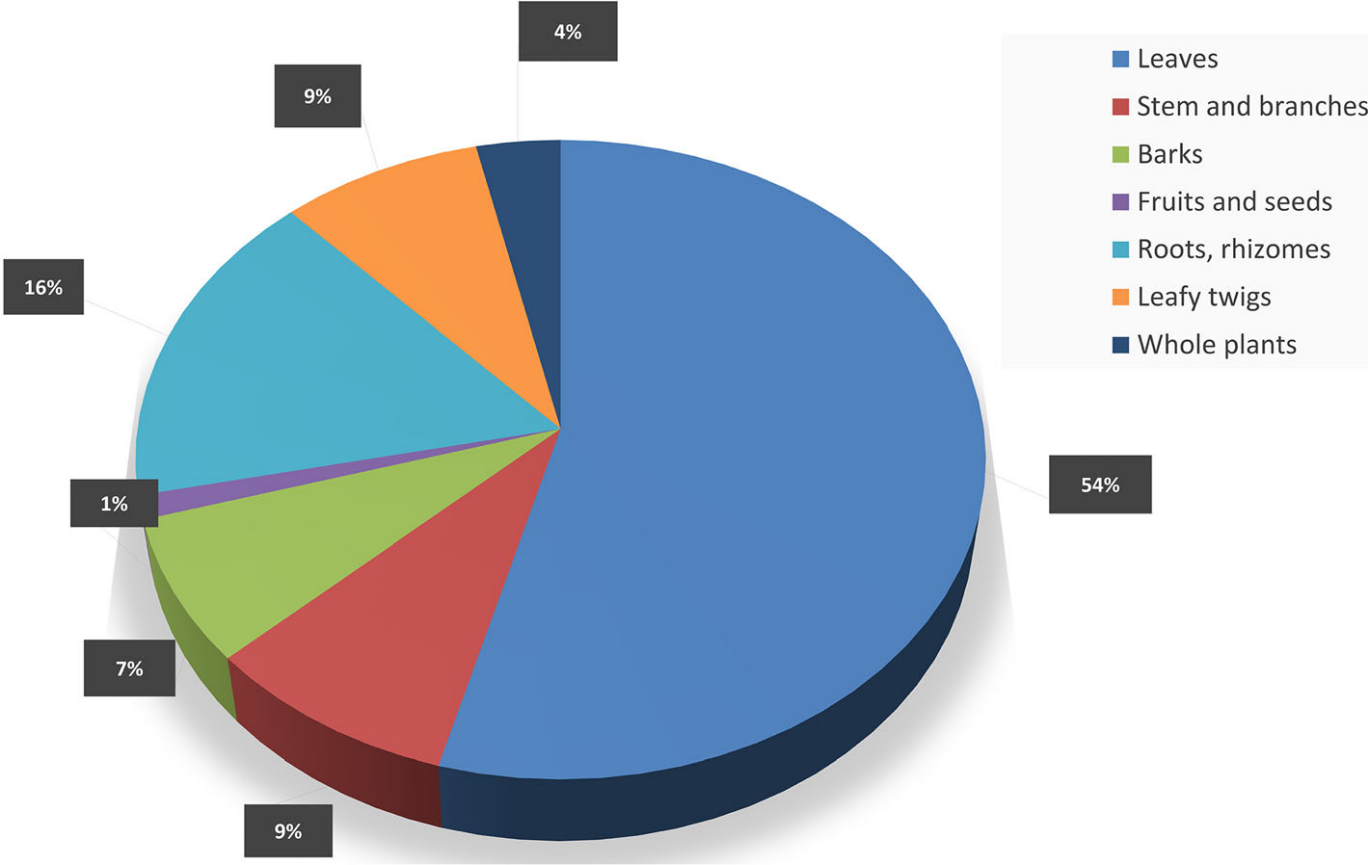
Percentage of medicinal plant parts used in the three communities in Sakaraha, South-western Madagascar

During our ethnobotanical surveys, we inventoried 235 taxa belonging to 75 families and 198 genera used as medicinal plants (Additional file 1). Fabaceae (25 taxa), Apocynaceae (14 taxa), Rubiaceae (14 taxa), Euphorbiaceae (11 taxa), and Asteraceae (10 taxa), were the plant families with the highest number of species (Fig. 2). Plant parts including root, rhizome, aerial parts, whole plant, fruit, seed, leaves, bud, and bark from trees, herb, shrub, liana and epiphytic plants are used. Leaves were found as the most used plant part (54%) while fruits and seeds were the least ones (1%) (Fig. 3).

Of all plants gathered during the study, 51% of the taxa were found in the dry forest surrounding villages, 13% were collected from the Analavelona sub-humid forest because the forest is mostly for its cultural aspect, 14% collected from the savannah, 13% were semi-domesticated and 9% were cultivated species (*Zea mays*, *Oryza sativa*, *Ipomea batatas*) or bought by local people in the market place for their medicinal uses (*Cinnamosma fragans, Zinziber officinale* and *Curcuma longa*). Among the medicinal plants taxa 53% were endemic of Madagascar.

Regarding the abundance of medicinal plants in their natural habitat, all of them are abundant but it appears that mature individuals of highly sought species such as *Cedrelopsis grevei* and *Vanilla madagascariensis* have become very hard to find. During our ethnobotanical surveys, respondents cited three medicinal plant species namely *Neobeguea mahafaliensis*, *Millettia richardiana* and *Helichrysum faradifani* have been hardly found in their area anymore.

### Preparation and administration mode of medicinal plants

**Fig. 4:**
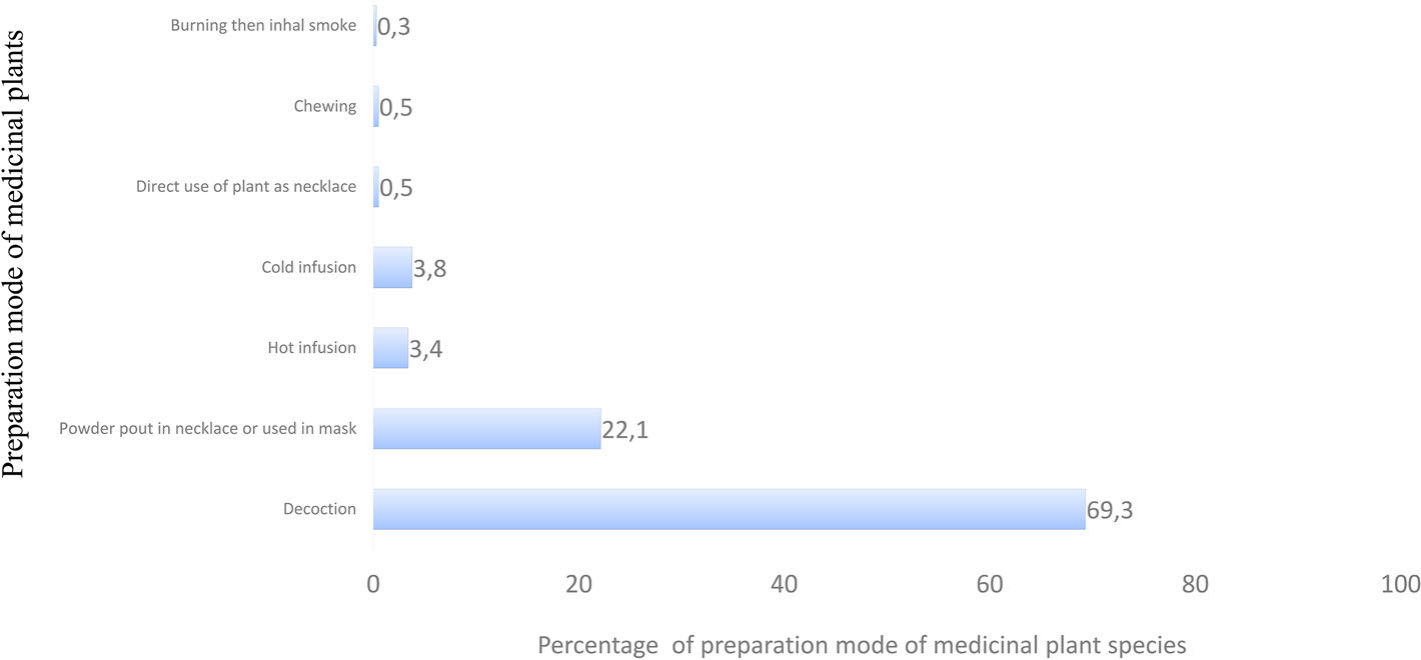
proportion of preparation mode of medicinal plant species in the three communities in Sakaraha, South-western Madagascar

Medicinal plants in the study area were prepared in many different ways depending on the species of plant itself, or its part used and the ailments treated be treated. In 51.5% of cases, medicinal plant species were prepared using more than one method. Decoction was the most used processes (69%) followed by the transformation of stem or root into powder (22%), and direct use of plants are rarely practiced (Fig. 4).

Regarding the administration mode, 75% of medicinal plants are taken orally as hot or cold drink, sometimes with sugar added, as mouthwash, and application on hard palate. External ways like bath, poultice and forehead mask are used for 24.8% of the cases.

### Frequency (Fq), Use Value (UV) and Fidelity Level (FL) of medicinal plants

The analysis of medicinal plant list showed that people living around Analavelona forest had a good knowledge of medicinal plants. In average each informant cited 6.7 ± 6.03 plants. More than 6 plants were cited by 40% of respondents, and 27% of them cited less than 4 plants. Medicinal plant species cited by more than two informants are considered as the most used medicinal plants are found (Additional file 2).

At individual species level, *Cedrelopsis grevei* was widely cited (Fq = 0.28) followed by *Henonia scoparia* (Fq = 0.25) and *Jatropha curcas* (Fq = 0.22). *Cedrelopsis grevei* and *Henonia scoparia* are the most used medicinal plants by local people with Use Value (UV) of 0.48 and 0.43; respectively, *Jatropha curcas* have lower Use Value (UV = 0.22) than *Woodfordia fruticosa* (UV = 0.29), *Tamarindus indica* (UV = 0.26), *Flacourtia ramontchi* (UV = 0.26) and *Phyllanthus casticum* (UV = 0.23).

The Fidelity Level of plants used in many categories of illness was high. A total of 40% (*n* = 94) of medicinal plants had high Fidelity Level (≥50%). Only 6.8% (*n* = 16) of species had the highest Fidelity Level (≥ 90%). Species with high Fidelity Level were mostly used for Digestive System Disorder (DSD), Infectious Diseases (ID), Pregnancy Birth and Puerperium (PBP) and Respiratory System (RespS).

### Medicinal plant knowledge among informants following the social status

For laypeople, there was no difference between men and women (*p* = 0.52) regarding to medicinal plant knowledge as in average, men cited 6.8 ± 1.4 and women cited 6.7 ± 7.07. Informants aged between 45 and 60 year-old cited the maximum number of species (9 ± 6.1) and the youngest ones (<15 year-old) cited the least medicinal plants species (3 ± 2) similar to the eldest ones (>75-year-old). Data analysis showed low correlation (r = 0.17, *p* = 0.013) between informants’ ages and their medicinal plants knowledge. No difference (*p* = 0.83) were found among medicinal plants cited by the six ethnic groups living in the study site. Data analysis showed no correlation (r = 0.04, *p* = 0.05) between educational level and the number of cited medicinal plants. Significantly higher average number of plants (*p* = 0.0084) was reported by informants considered as expert on plant uses and identification (12.7 ± 10.8) than by community members who are not plant user’s specialists even though they have knowledge on their uses (6.2 ± 4.7). However, medicinal plant species quoted by simple informant are much higher (208 taxa) than those cited by expert informants (144 taxa). Among the most cited species, 23 species such as *Aristolochia albida, Anacolosa pervilleana, Antidesma madagascariensis, Rhynchosia minima, Cardiospermum halicacabum, Aloe vahombe, Acacia bellula, Alchornea alnifolia, Cynanchum luteifluens var. longicoronae, Marsdenia verrucosa, Tridax procumbens, Carica papaya, Equisetum ramossissimum, Crotalaria retusa, Dalbergia bracteolata, Hazomalania voyronii, Ocotea trichantha, Ficus botryoides, Echinochloa colona, Gouania pannigera, Coffea perrieri, Paederia grandidier* and *Salvadora angustifolia*, were mentioned by simple informants only and *Acridocarpus excelsus* was cited exclusively by expert informants.

## Discussion

All previous ethnobotanical works led in other parts of Madagascar showed a high number of plant species used for medicinal purposes [2–4, 36, 37]. The present study conducted in Southwestern Madagascar is not an exception, we recorded 235 species of medicinal interests. The number of medicinal plants cited by informants in this study is higher when compared to previous study from the southern part of Madagascar. Jaccard index revealed no similarities (JI = 0.05) with medicinal plants listed by Debray et al. [35] and a weak similarity (JI = 0.17) with those reported by Gallée et al. [15] from St Augustin and Betsinjaky, Toliara II. Dissimilarities between studies showed high diversity of traditional knowledge in the southern Madagascar and underlined the importance of ethnobotanical data gathering efforts in the areas where nature is marginal and fragile.

We also found out that all illnesses encountered within three communities from Mahaboboka, Mikoboka, Amboronabo have been treated with plants material. However, strangely, we did not get or hear during our investigation any information about plant species that cure diseases that are known to be prevalent throughout the Southern Madagascar [15, 35]. For example, no members of the community mentioned plant species that treat vaginal or urinary infection, skin allergy or burn, leprosy, animal bites and hemorrhoids. These diseases belong to personal intimacy and hardly be disclosed to strangers. Interestingly, the four diseases, that are recognized by WHO as problematic for the Madagascar nation such as malaria, diarrhea, wound healing and dental health [38], were encountered in the site study. Among these diseases, malaria kept devastating the area, and is still the main cause of infant and children mortality all over the Sakaraha district. Despite of numerous ethnobotanical studies reporting studies on traditional healing of malaria [39–42], still no improved traditional medicines curing malaria are readily available in the local market.

Like numerous reports on medicinal plants in Madagascar [2, 4, 36, 43, 44], this study revealed also that Digestive System Disorders like diarrhea were the most cited by informants. In this study as in some study undertaken in Madagascar [2–4, 36], Dental Health are not relevant to informants. Tooth and mouth health was rarely cited by local people as they seem not suffering much of dental problems. The reason behind this may be the low sugar diet and milk consumption from zebu livestock which help to strengthen teeth [45, 46], but also the quality of water they use for their daily life, which is rich in Calcium [18].

Other conditions such as: pregnancy, birth and after birth and babies’ fontanel problem (late or early closing of babies’ fontanel) were also among the most treated. Babies’ fontanel problem are part of frequent diseases mentioned in this study and by Gallé et al. [15] in their work in the southern Madagascar but not much found in other studies [2–4, 36, 43, 44]. These diseases are mainly caused by acute dehydration [47], which occurs frequently in the southern Madagascar.

Values Informant Consensus Factor (Fic) of different use categories of illnesses from this study showed that Fic values of Pregnancy, Birth and Puerperium (PBP), Respiratory System and Cranial System categories were much higher than Fic value of the four illnesses (malaria, diarrhea, wound healing and dental problems) prioritized by the World Health Organization (WHO). This indicates that people had greater agreement for plants used to treat diseases related to pregnancy, child birth and child care after birth because Bara ethnic group give strong interests in procreation and preserving heritage. Our first hypothesis stated that people have greater agreement to the category of illnesses related to malaria, wounds, diarrhea and dentistry diseases is therefore rejected.

About medicinal plant species to treat those illnesses categories, more than 10 plant species were cited by the communities from Mahaboboka, Amboronabo and Mikoboka to cure the four most important diseases in Madagascar (malaria, wounds healing, diarrhea and dental problems) according to the World Health Organization [38]. More than 40% of informants agreed that three species (*Garcinia pauciflora*, *Moringa oleifera* and *Psiadia altissima* var. *occidentalis*) were used to treat malaria. Among these three species only *Moringa oleifera* was cited in other studies as traditionally used to cure malaria [48, 49]. However, they did not mention several species like *Tamarindus indica, Zanthoxylum tsihanimposa*, *Toddalia asiatica* fruits and root barks, and *Hazomalania voyronii*, that have been knownto have anti-malarial properties [40, 50–53], even though they occur in Analavelona forest and its surroundings. Perhaps communities living around Analavelona forest should be informed of the antimalarial properties of these species to help them fighting the Malaria epidemy.

Wound healing was treated by plant species which were uncommon to the usual list of medicinal plants from Madagascar. The two species *Maerua nuda* and *Erythroxylum pervillei* showed high fidelity level to heal infected wound. These two species are also proposed for further studies.

Like numerous reports on medicinal plants in Madagascar [2, 4, 36, 43, 44], this study revealed also that Digestive System Disorder like diarrhea were mostly cited by informants. The use of some species such as *Psidium guajava*, *Toddalia asiatica, Celtis gomphophylla*, *Senna occidentalis*, *Pulchea bojeri* [2, 15, 54–56] for Digestive System Disorder is widely common. Nevertheless, the use of species like *Strychnos henningsii, Bridelia pervilleana, Vernonia poissonii* and *Vitex lanigera* to treat diarrhea is new, and we suggested further pharmacological work to be conducted with these species.

Only three species were cited for dental problems. This is fewer than the 7 species found by Gallé et al. [15] in the southern part of Madagascar and the number of species recorded during the inventory of dental medicinal plants in Madagascar [57, 58].

Useful plants species to treat Pregnancy, Birth and after birth [59], Respiratory and Cranial systems problems were important for all communities. When compared to species reported by Gallé et al. [15], species having high fidelity level such as *Achyrocalyx decaryi*, *Gardenia rutembergiana*, *Allophylus cobbe* var. *dissectus* and *Rinorea greveana* for Respiratory System problem; *Woodfordia fruticosa, Tetrapterocarpon geayi, Strychnos madagascariensis, Acacia bellula* and *Dalbergia bracteolata* for Cranial System problem, were new and therefore good candidates for further pharmacological essays.

Several species were mentioned by local community for having aphrodisiac properties. Among them, three endemic species (*Cedrelopsis grevei*, *Vanilla madagascariensis* and *Neobeguea mahafaliensis*) were widely recognized for their aphrodisiac properties [15, 37, 52, 60–65].

Regarding to the abundance of medicinal plants in the wild, scarcity of some medicinal plant species such as *Cedrelopsis grevei. Dalbergia purpurescens* and *Anacolosa pervilleana* can be explained by the multiple uses of these species in the study site. Apart from being used as medicines they are also collected for construction, tools and cultural purpose [66]. Some medicinal plants species like *Vanilla madagascariensis* and *Neobeguea mahafaliensis* are over-collected for their medicinal uses even if we did not record any data about market of those plants species from the study site. Some species like *H*e*lichrysum faradifani* and *Millettia richardiana* are threatened in the area by its habitat losses due to fires.

Results of this study showed that men and women have very good knowledge on medicinal plants in all categories of diseases. The reason is that, in the study area, women learn about medicinal plants during their young age when they begin to care for their household and babies and men do collect these medicinal plants in the dry and the sub-humid forests. Our second hypothesis is not verified as women and men cited the same number of plant species in all categories. Comparable results were found among non–specialist Antanosy villagers during free-listing [67] and by Torres-Avilez et al. on the global level [68].

We also found that people from different age categories displayed a comparable medicinal plant knowledge because the eldest, e.g. head of villages (“*Lonaky*”) share medicinal plant knowledge to younger generation. Moreover, young girls and boys who are precociously involve in marital life displayed good knowledge of medicinal plants used during pregnancy, birth and after birth [59]. In contrast to that, studies undertaken in Ethiopia [69] and China [70] showed an interest loss on the use of medicinal plants among young people caused by the influence of modernization.

People from different ethnic group and different educational level shared the same interest to medicinal plants knowledge, this fact opposed to other studies conducted elsewhere [70, 71] that illiterate ones have more medicinal plant knowledge. Study undertaken with informants in different social status showed that use of medicinal plants is of interest for all people. Expert informants cited high number of medicinal plants in average but also more plants for cultural uses categories [72]. Simple informants quoted many taxa mainly classified as cultivated medicinal plants.

## Conclusions

The study showed that people living in the surroundings of Analavelona forest used various plant species as remedies for several ailments listed in the study area. Plants are the only available and accessible resources for first cares as health base centers are far from the villages. 235 taxa were cited by people for their medicinal uses, among them 124 taxa were the most used medicinal plants. These plants were indicated for 76 diseases classified in 13 categories. Plants used for women’s care during pregnancy, child delivery and for post-partum care, and for digestive system disorder were the most cited. Local people showed strong agreement on the use of plants for these two categories and for children’s care.

This study revealed also some new plants species having high fidelity level that could be used in further studies for the discovery of new medicines. Knowledge of medicinal plants in areas surrounding Analavelona forest is well transmitted orally from elders to youngers, from dominant ethnic group to immigrants and from illiterate people to school going and to the other members of society. This work is significant as it helps the conservation of medicinal plants knowledge and constitutes a written document for the next generation. Results of this study will ease decision making for the conservation of Analavelona forest. For the continuation of the project, local communities will be aware of known plants properties which exist in the area. They could benefit traditional knowledge they disclose to the scientific community especially regarding the discovery of new medicines.

## Additional files

Additional file 1: List of all medicinal plants encountered in the three communities (Mahaboboka, Amboronabo, Mikoboka) in South-western, Madagascar. (XLS 38 kb)
Additional file 2: List of the most used medicinal plants in the three communities (Mahaboboka, Amboronabo, Mikoboka) in South-western, Madagascar. UV: Use value, FL: Fidelity Level. BCVP: Blood and Cardio-Vascular Problem, CS: Cranial System, DH: Dental Health, DSD: Digestive System Disorder, GA: General Ailments, ID: Infectious Diseases, MSS: Muscular Skeletal System, NS: Nervous System, PBP: Pregnancy, Birth and Puerperium, ReprS: Reproductive System, RespS: Respiratory System, SS: Sensory System, Vet: Veterinary. L: Leaves, YL: Young Leaves, Lt: Leafy twigs, S: Stem, YS: Young Stem, La: Latex, B: Bark, W: Whole plants, R: Roots, Rh: Rhizome, Bu: Buds, Fr: fruits, Se: Seeds. (XLS 98 kb)
